# Enhancing Adolescent Physical Fitness and Well-Being: A School-Based High-Intensity Interval Training Program

**DOI:** 10.3390/jfmk9040279

**Published:** 2024-12-20

**Authors:** Petar Mitić, Rade Jovanović, Nikola Stojanović, Valentin Barišić, Nebojša Trajković

**Affiliations:** 1 Faculty of Sport and Physical Education, Department of Theoretical-Methodological Subjects, University of Niš, 18000 Niš, Serbia; mitic.petar@gmail.com (P.M.); radejovanovic75@gmail.com (R.J.); nikola987_nish@hotmail.com (N.S.); 2 Faculty of Medicine, Physical Education Department, University of Niš, 18000 Niš, Serbia; 3 Faculty of Kinesiology, University of Zagreb, 10000 Zagreb, Croatia; valentin.barisic@kif.unizg.hr

**Keywords:** well-being, classes, physical health, young, intervention

## Abstract

**Background**: The aim of this study is to investigate the effects of a school-based high-intensity interval training (HIIT) program on quality of life and physical fitness in adolescents. **Methods**: The study included 60 male adolescents (16.2 ± 0.6 years), randomly assigned to one of two groups: the HIIT group (n = 30) and the control group (CG, n = 30). Participants completed the KIDSCREEN-27 questionnaire to evaluate quality of life and physical fitness tests, including vertical jump, repetitive strength, agility, and flexibility. The experimental program consisted of a 12-week intervention integrated into the warm-up phase of regular physical education (PE) classes. Each PE class lasted 45 min, with the HIIT regimen occupying 10 min of the warm-up phase. The HIIT sessions followed a Tabata-style structure, performed twice weekly. A two-way ANOVA with repeated measures assessed group and time interaction effects. **Results**: The results revealed changes in physical well-being following the HIIT intervention (*p* = 0.01) compared to the control group (*p* = 0.42). The results of the two-way ANOVA with repeated measures revealed interaction effects between group and time for the various fitness tests, countermovement jump (*p* = 0.04), 4 × 10 m agility test (*p* = 0.02), medicine ball throw test (*p* = 0.04), and sit-ups (30 s) (*p* = 0.01). **Conclusions**: This study highlights the effectiveness of school-based HIIT in significantly enhancing physical well-being and physical fitness among adolescents.

## 1. Introduction

The inactivity of the population represents a health risk because it can negatively affect both mental and physical health and, consequently, the economy and society [1]. Quality of life and physical fitness are essential for adolescents. They significantly contribute to mental health, academic performance, and long-term health outcomes, helping adolescents develop resilience and healthy lifestyle habits into adulthood [2,3]. According to Pires-Júnior et al. [4], there is a significant positive association between physical fitness and health-related quality of life in adolescents, with higher fitness levels contributing to enhanced physical, social, and emotional well-being, collectively improving overall quality of life. International guidelines recommend that all children and adolescents complete at least 60 min of moderate to vigorous physical activity daily to ensure full health benefits [5,6]. Unfortunately, most adolescents do not meet the abovementioned recommendations [7].

Physical activity supports physical fitness and mental health, enhances quality of life, and boosts adolescent motivation [8]. Regular exercise has been linked to improvements in mood, reductions in symptoms of depression and anxiety, and increased stress resilience, contributing to a higher quality of life [9]. It promotes physical health and influences psychological well-being and social cohesion, which are particularly important during adolescence, which is marked by significant emotional and social development [10]. To assess health-related quality of life (HRQoL) in this population, the KIDSCREEN-27 questionnaire has been widely validated across various cultural contexts, including Serbia. This tool evaluates multiple dimensions of well-being, making it highly appropriate for capturing the potential impact of physical activity interventions [11,12]. Additionally, physical activity offers adolescents an outlet to experience a sense of accomplishment, socialize, and build resilience, all of which are key motivators for sustained participation in exercise [13].

Various forms of exercise, particularly engaging and team-oriented, have positively impacted adolescents’ quality of life [9,10,13]. Programs that emphasize high-intensity and enjoyable activities, such as school-based sports or team-based physical education, foster both physical and mental health benefits by promoting social connections and providing structured physical challenges [14]. High-intensity interval training (HIIT), in particular, has shown promise for improving physical and mental health due to its efficiency and adaptability [8].

Given the insufficient provision of physical education classes in Serbian high schools, where students typically have only two 45 min sessions per week, implementing brief, high-intensity interventions is practical and necessary. Evidence indicates that even short-duration high-intensity training can yield meaningful fitness improvements in adolescents. For instance, Koźlenia et al. [15] demonstrated that high-intensity functional training (HIFT) with progressively increasing session durations ranging from 6 to 14 min significantly improved adolescent muscle mass and strength. Building on this evidence, our study employed 10 min HIIT sessions designed to provide an efficient and scalable solution within the constraints of school schedules, ensuring that PE classes retain their broader curricular goals while integrating effective fitness interventions. This approach reflects the potential for schools to play a central role in promoting adolescent fitness through structured, time-efficient programs.

Schools play a critical role in implementing exercise programs that are both high in intensity and engaging, which is essential to improving quality of life and promoting sustained physical activity [14,16]. Integrating HIIT into school curricula can help adolescents maintain motivation by providing diverse, high-energy experiences that align with their social and physical needs [17]. As research suggests, these structured, enjoyable, and high-intensity programs have the potential to make exercise a valued part of adolescents’ lives, supporting both their physical and mental well-being in meaningful ways [8,16].

Enjoyment is crucial for children and adolescents’ participation in exercise, as it fosters a positive attitude toward physical activity and encourages consistent engagement [14]. Adolescents who enjoy exercise are more likely to participate regularly, resulting in sustained benefits for their physical and mental well-being [2]. HIIT has been shown to generate positive affective responses and enjoyment among adolescents, making it an appealing exercise format for this age group [18]. The authors also stated that adolescents may find HIIT particularly enjoyable due to its short bursts of intense activity followed by brief rest periods, which can be more stimulating and engaging than traditional continuous exercise [18]. Given these benefits, incorporating HIIT into school-based programs can provide a fun, dynamic, and effective way to promote regular physical activity among adolescents, helping them to build lifelong healthy habits through enjoyable and impactful exercise.

While HIIT has been widely researched for its effectiveness in improving physical fitness, cardiometabolic health, and athletic performance across various age groups, there is limited evidence regarding its effects on psychosocial outcomes, particularly in adolescents. This age group, which experiences unique physical and emotional developmental challenges, often faces barriers to maintaining consistent physical activity, which impacts their quality of life and intrinsic motivation to exercise. Although some studies suggest that HIIT may positively impact mental health markers in adults, the extent to which these findings apply to adolescents is unclear. Furthermore, the existing research rarely addresses how the demanding nature of HIIT might influence adolescents’ quality of life. Therefore, this study aims to investigate the effects of a structured HIIT program on quality of life and physical fitness in adolescents.

## 2. Materials and Methods

### 2.1. Participants

The study sample comprised 60 male adolescents (16.23 ± 0.6 years) enrolled at a secondary grammar school. Participants were randomly assigned to one of two groups: the high-intensity interval training (HIIT) group and the control group (CG). Each group comprised of 30 subjects. General descriptive parameters for both groups can be seen in Table 1. The randomization process was executed using standard computer software following the initial testing phase. Inclusion criteria stipulated that participants were male adolescents within the specified age range who were not engaged in organized training programs during the study. Furthermore, all participants were required to present a valid medical certificate confirming their health status and ability to participate in the prescribed activities. Exclusion criteria included individuals with respiratory or cardiovascular diseases, developmental disabilities, or chronic illnesses, those recovering from injuries or illnesses, and any individuals currently participating in competitive sports. Before the onset of the study, the participants’ parents and the grammar school principal gave their written approval. The participants were allowed to withdraw from the experimental treatment at any time. In addition, the participants and their parents were acquainted with the importance and advantages of this study. The Ethical Committee of the Faculty of Sport and Physical Education, University of Niš, has approved this research. This study was conducted following the Declaration of Helsinki, as part of a doctoral dissertation, the topic of which was adopted at the University of Niš, Serbia (decision No. 8/18-01-007/23-031, approval date 6 November 2023).

### 2.2. Testing Procedures

The testing procedures involved several key steps to assess participants’ quality of life and physical fitness. All assessments were conducted in a controlled environment to ensure consistency and reliability of the results. Testing was scheduled for the morning to minimize variations in physical activity levels that could affect the outcomes. The first step involved measuring anthropometric data, including height, weight, and body mass index (BMI). These measurements provided baseline anthropometric data to contextualize participants’ physical characteristics and fitness levels. After anthropometric assessments, participants completed the KIDSCREEN-27 questionnaire to evaluate their quality of life. This structured questionnaire captures various dimensions of well-being, enabling researchers to assess the impact of physical activity on participants’ perceived quality of life. Finally, physical fitness assessments were conducted, which included tests such as vertical jump, repetitive strength, agility, and flexibility. These measures provided objective data on the physical fitness levels of the participants, allowing for a comprehensive evaluation of the effects of the intervention. Before fitness testing, a standardized warm-up included dynamic stretches and mobility exercises designed to prepare the body for physical activity, increase heart rate, and improve range of motion. Warm-up consisted of light jogging, high knees, butt kicks, arm circles, and leg swings, lasting approximately 10 min, to enhance performance and reduce the risk of injury during subsequent fitness assessments.

To measure body height and body mass, and calculate body mass index (BMI), valid instruments such as the Seca 217 stadiometer and Seca 874 electronic scale (Seca, Hamburg, Germany) were employed. To measure body height, participants stood barefoot with their backs to the stadiometer and heels together, ensuring an upright posture. At the same time, the headpiece was lowered to the crown of the head, recording the height in centimeters to the nearest 0.1 cm. Participants removed shoes and heavy clothing for body mass, standing centrally on the scale until the reading stabilized, which was recorded in kilograms to the nearest 0.1 kg. BMI was then calculated using the formula BMI = weight (kg)/(height (m))^2^, converting height from centimeters to meters by dividing by 100.

### 2.3. Quality of Life

KIDSCREEN-27 is a health-related quality of life (HRQoL) questionnaire designed specifically for children and adolescents [11]. It consists of 27 items that assess various dimensions of well-being, including physical, psychological, and social aspects of health. The questionnaire is divided into five factors: Physical Well-Being, Psychological Well-Being, Autonomy and Parent Relations, Social Support and Peers, and School Environment. Each item is rated on a 5-point Likert scale, allowing respondents to express their level of agreement with various statements about their health and quality of life.

The KIDSCREEN-27 questionnaire has been validated in multiple cultural contexts, demonstrating its reliability and validity as a measurement tool for quality of life among children and adolescents. For instance, Budler et al. [19] conducted a translation and validation study of the Slovenian version of KIDSCREEN-27, confirming its applicability and reliability for the Slovenian population. Similarly, Stevanović et al. [12] evaluated the Serbian version, establishing its reliability, validity, and agreement between children’s and parents’ ratings.

### 2.4. Enjoyment

The Physical Activity Enjoyment Scale (PACES) assesses how much adolescents in the HIIT and control groups enjoyed their physical activity experience. Adapted from Abassi et al. [20], the scale consists of 18 items rated on a 7-point bipolar scale, which participants completed after the intervention to capture their immediate feelings about the exercise. The questions covered various aspects of enjoyment, with instructions prompting participants to reflect on their physical activity experience at that moment. An overall enjoyment score was calculated by summing the scores from each item, ranging from 18 to 126. Higher scores on this scale indicate greater enjoyment, which provided insights into the adolescents’ subjective responses to HIIT versus the control activities.

### 2.5. Physical Fitness

#### 2.5.1. Vertical Jump

The countermovement jump (CMJ) and the squat jump (SJ) are commonly used tests for assessing lower body power and explosive strength. Both tests can be effectively measured using the Opto Jump system (Microgate, Bolzano, Italy), which records jump height based on flight time. In the CMJ, participants begin in an upright position, perform a downward squat, and then jump upward explosively, utilizing the stretch-shortening cycle for enhanced performance. In contrast, the SJ starts from a static squat position, and participants jump upward without a preparatory downward movement. Both tests have demonstrated good validity and reliability across different populations, making them valuable tools for evaluating athletic performance and training effects [21].

#### 2.5.2. The 4 × 10 m Agility Test

Using a stopwatch, the 4 × 10 m agility test assesses an individual’s speed and agility. Participants start from a standing position and sprint 10 m, turn around a cone, return 10 m, and repeat this sequence four times. The total time taken to complete the course is recorded. This test has shown good validity and reliability for evaluating agility performance [22].

#### 2.5.3. Sit and Reach

The Sit and Reach test is commonly used to assess general flexibility, primarily focusing on the hamstrings and lower back. During the test, participants sit on the floor with their legs extended and feet placed against the Sit and Reach box. They are instructed to reach forward as far as possible while keeping their knees straight, and the maximum distance reached (in cm) is recorded. Despite its widespread use across various populations, the test primarily provides a general measure of flexibility rather than isolating specific muscle groups [22].

#### 2.5.4. Medicine Ball Throw

The lying medicine ball throw test, as outlined by Palao and Valdes [23], is a reliable method for assessing upper-body strength and power. In this test, participants lie supine (on their back) on a stable surface, such as a mat or bench, with their knees bent to maintain stability. A 3 kg medicine ball is used for the test. The participant holds the medicine ball with both hands at chest level and, from a stationary position, explosively throws the ball upwards as far as possible.

To ensure proper execution and participant safety, participants received detailed instructions and a demonstration of the correct technique before testing to minimize the risk of injury. The testing area was cleared of obstructions, and only the participant and the test administrator were present to prevent distractions. Additionally, a spotter was positioned nearby to assist with retrieving the medicine ball and to provide oversight during the test. The weight of the medicine ball (3 kg) was verified to be appropriate for the participant’s age and physical condition, avoiding excessive strain.

During the test, the participant’s back was required to remain in contact with the ground throughout the throw, and the movement was initiated explosively from the chest. The distance the ball traveled was recorded to measure upper-body power and strength. This protocol provided a controlled and safe environment for evaluating upper-body power output, particularly targeting the pectoral and triceps muscles, and has been validated for reliability and accuracy in previous studies [23].

#### 2.5.5. Sit-Ups for 30 s

The sit-up test for 30 s is designed to measure core strength and endurance. During the test, participants lie supine with knees bent, feet unanchored on the floor, and hands placed behind the head, following a standardized protocol for abdominal exercises. On the tester’s command, participants perform as many complete sit-ups as possible within 30 s, aiming to reach their maximum repetition count in the allotted time. Each sit-up should be executed with controlled movement, lifting the upper body until the elbows touch the knees and then returning them fully to the mat to count as one repetition. The final score is the total number of sit-ups completed within 30 s, with the test emphasizing both strength and endurance in the abdominal muscles [15]. This test is widely used due to its simplicity and effectiveness in quickly assessing core fitness levels.

### 2.6. Exercise Program

**Figure 1:**
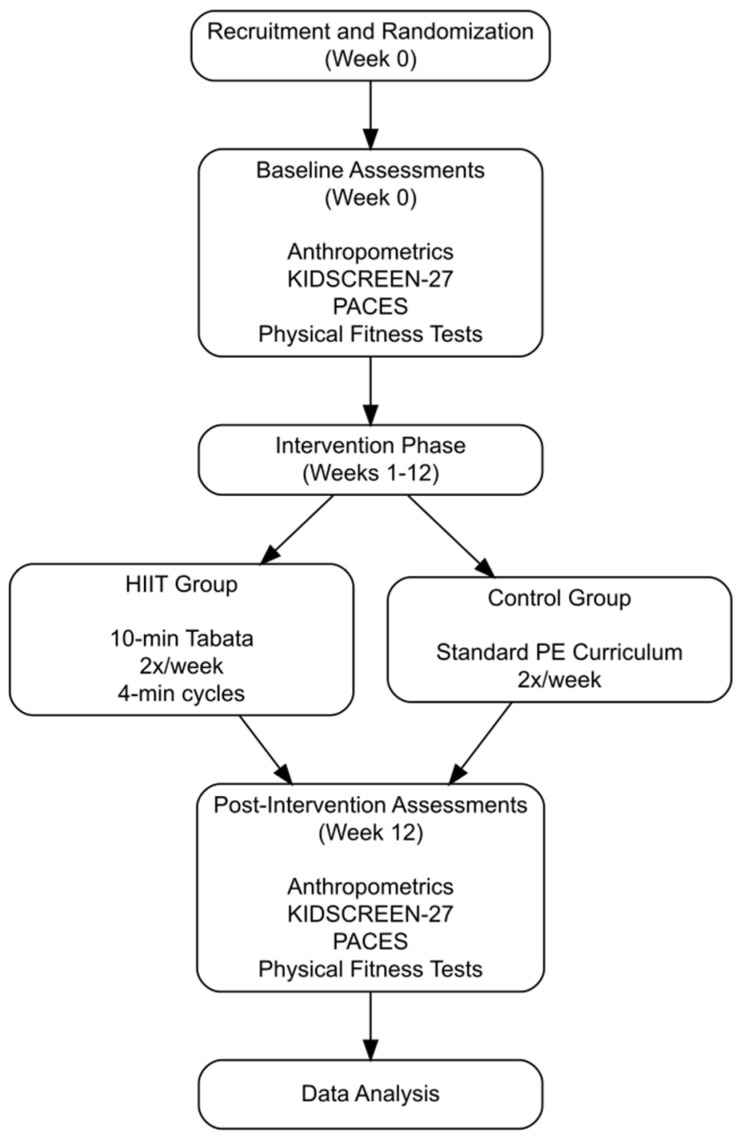
Study timeline: recruitment, intervention, and assessment phases.

The experimental component of this study was structured as a 12-week HIIT program specifically tailored for high school students (Figure 1). This program was integrated into the four-phase structure of regular physical education (PE) classes, held twice weekly for 45 min. Each PE class consisted of the following phases: (1) a five-minute general warm-up performed by both groups; (2) a 10 min preparatory phase, during which the experimental group engaged in a Tabata-style HIIT regimen, while the control group performed standard preparatory activities outlined in the PE curriculum; (3) a 20 min main phase, in which both groups participated in the same curriculum activities; and (4) a 10 min cool-down phase completed together by both groups.

The HIIT intervention lasted for a total of 10 min during the preparatory phase of the class. The experimental group engaged in two Tabata sessions, each lasting four minutes. Each Tabata session comprised eight exercises (wall ball, burpees, split jumps, jumping jacks, push-ups, crunches, frog jumps, and Russian twists), with each exercise performed for 20 s, interspersed with 10 s of rest. Additionally, a one-minute rest period was implemented following each complete Tabata cycle. Meanwhile, the control group engaged in standard preparatory exercises, such as dynamic stretching and light aerobic activities.

To ensure the desired intensity, the program was designed to elicit a heart rate corresponding to 80–90% of each participant’s maximum heart rate (HRmax). HRmax was determined through a shuttle run protocol, which is a validated method for assessing cardiovascular capacity. During the HIIT sessions, participants’ heart rates were continuously monitored using wrist-based heart rate monitors (Garmin Forerunner 245; Garmin Ltd., Taiwan). The devices provided real-time feedback, and instructors supervised each session to ensure adherence to the target heart rate range. If a participant’s heart rate fell below 80% HRmax, verbal cues were given to encourage increased effort to achieve the desired intensity. Conversely, if a participant exceeded the prescribed HRmax (over 90%), they were instructed to decrease the intensity of their movements or take brief pauses to allow their heart rate to return to the target range. This protocol ensured consistent exercise intensity while prioritizing participant safety and the efficacy of the intervention.

The control group exclusively participated in the standard activities outlined in their PE curriculum (Figure 1), allowing for a clear comparison of the effects of the HIIT intervention on quality of life and fitness outcomes.

### 2.7. Statistical Analysis

Data were analyzed using SPSS software (SPSS v.23, IBM Corporation, New York, NY, USA), and normality and sphericity assumptions were tested before conducting the ANOVA. A T-test for independent samples was conducted to check for group differences at baseline. The statistical analysis for the study comparing the effects of HIIT and a CG utilized a two-factor ANOVA with repeated measures to assess the impact of the intervention on various outcome measures over time. This analysis allowed for examining within-group changes across pre- and post-intervention time points and between-group differences in response to the intervention. Specifically, the factors included group (HIIT vs. CG) and time (pre-test vs. post-test), providing a comprehensive view of the interaction effects. Post hoc analyses were performed using Bonferroni corrections to determine where significant differences existed, when applicable. The significance level was set at *p* < 0.05 for all tests. Partial eta squared (η^2^) is reported as a measure of effect size and classified as small (0.01), medium (0.06), and large (0.14), according to Cohen [24].

## 3. Results

**Figure 2:**
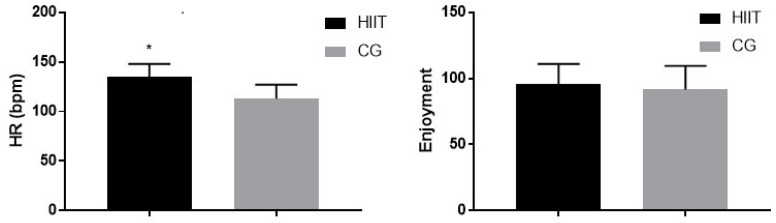
Mean heart rate and enjoyment levels during PE classes for the HIIT and control groups. The left panel displays the average heart rate (bpm) recorded during the entire 45 min PE classes, including a 10 min Tabata-style HIIT session for the experimental group (HIIT) and standard preparatory activities for the control group (CG). Heart rate was continuously monitored throughout the 45 min PE class, and the average values are presented. The right panel presents PACES enjoyment scores for both groups. The asterisk (*) indicates a significant between-group difference. Error bars represent standard deviations.

Figure 2 illustrates the mean heart rate (HR) and enjoyment levels recorded during the 45 min PE classes for the HIIT and control groups. The average heart rate for the HIIT group (135.2 ± 13.4 bpm) was significantly higher than that of the control group (113.3 ± 14.8 bpm; *p* = 0.01), reflecting the elevated intensity of the 10 min Tabata session integrated into the HIIT group’s preparatory phase. As measured by PACES scores, enjoyment levels revealed no significant difference between the HIIT group (95.72 ± 15.41) and the control group (91.73 ± 17.81; *p* = 0.35).

The results revealed changes in physical well-being following the HIIT intervention (*p* = 0.01) compared to the control group (*p* = 0.42) (Table 2). In the HIIT group, the mean score for physical well-being improved from a pre-test mean of 18.23 (SD = 3.40) to a post-test mean of 19.17 (SD = 2.94), indicating an increase in physical well-being following the intervention. The control group had a smaller improvement, with the pre-test mean at 19.53 (SD = 2.76) and the post-test mean at 19.87 (SD = 2.34). The analysis of the remaining KIDSCREEN-27 variables—Psychological Well-Being, Autonomy and Parents, Peers and Social Support, and School Environment—showed no statistically significant changes (*p* > 0.05) within either the HIIT or control groups from pre-test to post-test.

The results for the physical fitness variables, including the countermovement jump (CMJ), squat jump (SJ), 4 × 10 m agility test, Sit and Reach (SAR) test, medicine ball throw, and sit-ups (30 s), indicated significant time effects (*p* < 0.05), showing improvements in most physical performance tests across both the HIIT and control groups from pre- to post-test (Table 3). The results of the two-way ANOVA with repeated measures revealed interaction effects between group and time for the various fitness tests. For the CMJ test, a significant interaction effect between time and group was observed (F = 4.20; *p* = 0.04), with a large effect size (η^2^ = 0.60). In the 4 × 10 m agility test, a significant time × group interaction effect was observed, F = 5.75 *p* = 0.02, with a very large effect size (η^2^ = 0.90). The medicine ball (MB) throw test also showed a significant time × group interaction effect (F = 4.41 *p* = 0.04), with a large effect size (η^2^ = 0.71). Finally, the sit-ups (30 s) test revealed a statistically significant interaction effect, F = 9.01 *p* = 0.01, although with a smaller effect size (η^2^ = 0.13). However, no significant time x group interactions (*p* < 0.05) were observed for other fitness performance tests.

## 4. Discussion

This study aimed to investigate the effects of a HIIT program implemented in physical education classes on adolescents’ quality of life and physical fitness. The results indicated that while the HIIT group significantly improved physical well-being compared to the control group, no significant differences were observed in other quality-of-life dimensions, such as psychological well-being, autonomy, or social support. Regarding physical fitness, the HIIT group exhibited greater improvements in lower-body power (CMJ, SJ), agility (4 × 10 m test), upper-body power (medicine ball throw), and core endurance (sit-ups test) compared to the control group. These findings highlight the potential of integrating short, high-intensity protocols within physical education settings to improve specific physical fitness components while positively influencing physical well-being.

Our study demonstrated that integrating a school-based HIIT program into physical education classes significantly improved physical well-being in adolescents, aligning with previous research highlighting the benefits of physical activity interventions for promoting health and vitality [25]. A recent systematic review and meta-analysis further supports this finding, reporting small but significant improvements in health-related quality of life, particularly in physical well-being, due to physical activity interventions [26]. Similarly, school-community-linked programs have shown effectiveness in improving health-related quality of life, suggesting that integrating physical activity into structured environments can have positive impacts [27]. However, we did not observe significant changes in other quality-of-life dimensions, such as psychological well-being or social support. This variability aligns with findings from the Active Smarter Kids (ASK) study, which showed that not all school-based interventions lead to broad quality-of-life improvements [28]. These differences may stem from intervention design, duration, or participant engagement variations. For instance, while our study emphasized physical fitness improvements through high-intensity training, additional strategies, such as interventions targeting social and psychological dimensions, may be required to achieve more comprehensive improvements in quality of life. Our findings contribute to the growing body of evidence suggesting that targeted, context-specific interventions can effectively improve select aspects of quality of life in adolescents, particularly physical well-being.

HIIT typically involves short bursts of intense exercise followed by brief recovery periods, making it efficient and potentially engaging for youth. Studies indicate that HIIT may positively influence adolescents’ quality of life, particularly through improvements in physical health, mental well-being, and self-esteem, as these changes often result from increased physical fitness and decreased body fat levels [29,30].

Although physical fitness and body composition improvements are well-documented in adolescent HIIT interventions, the effects on quality of life are mixed and sometimes modest. For instance, Costigan et al. [29] observed that HIIT led to increased self-reported physical and social well-being among adolescents, aligning with findings that suggest physical fitness improvements can enhance self-perception and social interactions. Other research notes that while HIIT improves physical parameters, it does not consistently translate to significant changes in quality of life [31]. These outcomes may depend on the specific population, the duration and frequency of HIIT sessions, and the intensity of the exercises. The current results showed that the HIIT group experienced significant improvements in physical well-being compared to the CG. However, it is important to interpret these findings cautiously, as the limited intervention duration and focus on physical outcomes may not fully capture the broader dimensions of quality of life.

Our HIIT program may have shown a significant improvement in physical well-being but not in other dimensions of quality of life for several potential reasons. First, HIIT is highly effective for improving physical fitness markers such as cardiorespiratory endurance, muscle strength, and body composition, directly impacting physical well-being [29]. Nonetheless, the program’s brevity and intensity may have constrained opportunities for engagement and enjoyment, which are critical for broader quality-of-life enhancements in adolescents [32]. Furthermore, quality of life improvements in areas like emotional or social domains often require a more holistic approach, including social support and motivational elements that may not be intrinsic to a HIIT-only program [2]. Adolescents may benefit more from various exercise types, including moderate, steady-state activities that promote relaxation and stress reduction, which are closely tied to psychological well-being improvements [33]. Lastly, the short intervention period could be another factor, as broader quality-of-life changes may need longer durations. In contrast, physical changes are often measurable sooner [31]. Future research should explore these multidimensional approaches to fully understand the potential of school-based exercise programs for holistic quality-of-life benefits.

The HIIT group showed significant gains in agility, upper-body power, lower-body power, and repetitive strength, as indicated by performance improvements in the CMJ, 4 × 10 m agility, MB throw, and sit-ups (30 s) test. In recent years, school-based HIIT interventions have demonstrated promising effects on physical fitness in adolescents [34,35]. In addition, as reviewed by Logan et al. [36] and Costigan et al. [29], school-based HIIT interventions offer a practical solution for enhancing fitness levels within limited school time, supporting aerobic and anaerobic development through exercises easily adapted to school settings. Specifically, Duncombe et al. [34] and Bauer et al. [35] highlight the wide applicability of HIIT programs in school environments and their effectiveness in promoting physical activity and improving fitness levels among children and adolescents. Furthermore, school-based HIIT has improved cardiovascular and muscular fitness, agility, and explosive power [37]. These improvements, however, should be contextualized within the program’s constraints, as the relatively small effect sizes suggest the need for longer or more frequent sessions to maximize fitness gains.

The authors stated that these gains in physical fitness likely stem from the intensive nature of HIIT, which increases both muscular endurance and neuromuscular efficiency. Eddolls et al. [38] emphasize that integrating HIIT into school settings addresses physical fitness and can be an engaging way to meet recommended activity levels. These findings underscore the potential of school-based HIIT to boost physical performance in adolescents, making it a valuable addition to traditional physical education programs. The current findings on the positive effects of HIIT on physical fitness components align with the results reported by Jurić et al. [39], who also implemented HIIT during warm-up periods in physical education classes. Both studies observed significant improvements in fitness outcomes, particularly cardiovascular fitness, muscular strength, and overall physical performance. Jurić et al. [39] highlighted notable enhancements in students’ fitness levels attributed to the structured nature of the HIIT sessions embedded within the regular PE curriculum. The comparison of our results with those of Jurić et al. [39] is particularly relevant, because both studies utilized a similar framework for integrating HIIT into existing class structures. While we found improvements across various fitness tests, such as the CMJ and 4 × 10 m agility test, Jurić et al. [39] reported comparable gains, suggesting that this training modality effectively fosters fitness improvements among adolescents. However, it is important to acknowledge that the observed gains, while significant, may be influenced by the intensity and type of exercises, which could limit direct comparisons with studies employing different protocols. These school-based HIIT programs represent an accessible, impactful strategy for improving physical fitness outcomes among adolescents in structured educational settings.

While this study provides valuable insights into the effects of HIIT on physical fitness among adolescents, it is important to acknowledge several limitations. First, the study sample consisted exclusively of males, which limits the generalizability of the findings to the broader adolescent population, including females. Gender differences can influence responses to physical training interventions, and including a more diverse sample in future studies would provide a more comprehensive understanding of HIIT’s effects across different genders.

Additionally, the study’s relatively small sample size may have constrained the statistical power and the ability to detect subtle effects. Future research should consider larger, stratified samples to ensure that findings are robust and applicable across various subgroups. This approach could enhance the reliability of conclusions drawn from the data and help identify any differing impacts of HIIT on distinct demographics. Moreover, comparing the effects of HIIT with other training modalities, such as small-sided games, would be beneficial. Such comparisons could elucidate whether HIIT is more effective than other forms of training in improving quality of life outcomes among adolescents, providing clearer guidance for educators and coaches on the most effective training strategies.

Another limitation pertains to the flexibility assessment using the Sit and Reach test. While this test is commonly used, it primarily provides a general measure of flexibility rather than isolating specific muscle groups such as the hamstrings or lumbar spine. Furthermore, its results can be influenced by individual differences in limb and trunk proportions, potentially affecting the validity of the measurements. Future studies may benefit from employing alternative flexibility assessments, such as goniometry or motion analysis, to provide more accurate and specific flexibility evaluations.

Finally, although BMI was used to describe general anthropometric characteristics, its inability to distinguish between fat and muscle limits its relevance for comprehensively assessing health status. Since body composition can significantly influence physical well-being, which is a key component of quality of life, future studies should incorporate more precise measures, such as body fat percentage, waist-to-height ratio, or relative fat mass. These measures would provide deeper insights into the relationship between physical fitness, body composition, and quality of life.

## 5. Conclusions

The HIIT intervention demonstrated small-to-moderate improvements in specific physical fitness parameters, such as agility and lower-body power, while enhancing physical well-being. However, these effects were observed alongside gains in the control group, highlighting the overall influence of structured physical education programs. Broader quality-of-life dimensions did not significantly change, indicating that future interventions may benefit from more holistic approaches. Further research is needed to explore HIIT’s long-term impact, optimal duration, and intensity in diverse adolescent populations. This study underscores the potential of HIIT as a supplementary tool within existing school curricula to enhance physical fitness.

## Figures and Tables

**Table 1:** Basic anthropometric characteristics in HIIT and CG in pre-and post-testing.

HIIT			CG	
Outcome	Pre	Post	Pre	Post
Height (cm)	175.66 ± 8.77	176.66 ± 7.78	175.56 ± 5.71	176.26 ± 6.74
Body mass (kg)	66.73 ± 14.41	67.26 ± 13.38	71.09 ± 13.61	72.50 ± 13.63
BMI (kg/m^2^)	21.51 ± 3.67	21.66 ± 3.41	23.01 ± 3.99	23.34 ± 3.97

BMI—body mass index.

**Table 2:** Quality of life in HIIT and CG.

	HIIT (n = 30)			CG (n = 30)		
Kidscreen-27	Pre	Post	*p*	Pre	Post	*p*
Physical Well-Being *	18.23 ± 3.40	19.17 ± 2.94	0.01	19.53 ± 2.76	19.87 ± 2.34	0.42
Psychological Well-Being	26.10 ± 5.38	26.97 ± 5.99	0.14	27.73 ± 4.41	27.77 ± 3.96	0.93
Autonomy and Parents	30.87 ± 3.39	31.10 ± 3.85	0.71	30.53 ± 2.80	30.47 ± 3.50	0.80
Peers and Social Support	17.90 ± 3.17	17.93 ± 3.26	0.84	17.37 ± 2.34	17.13 ± 2.80	0.83
School Environment	12.77 ± 3.51	12.50 ± 3.52	0.45	14.17 ± 2.63	13.77 ± 3.64	0.44

* significant difference between HIIT and CG, *p* < 0.05.

**Table 3:** Differences in physical fitness between HIIT and CG.

	HIIT (n = 30)			CG (n = 30)		
Outcome	Pre	Post	*p*	Pre	Post	*p*
CMJ	27.95 ± 8.34	29.80 ± 7.69	0.001	31.70 ± 6.17	32.45 ± 5.38	0.015
SJ	23.64 ± 7.28	25.94 ± 6.69	0.001	25.07 ± 4.87	25.89 ± 5.31	0.06
4 × 10 m	12.27 ± 1.18	11.37 ± 1.05	0.001	11.86 ± 0.94	11.40 ± 1.13	0.001
SAR	3.62 ± 8.73	2.57 ± 7.68	0.6	2.20 ± 8.52	3.15 ± 8.59	0.7
MB throw	338.90 ± 97.98	348.88 ± 97.71	0.001	347.30 ± 93.34	351.03 ± 93.62	0.01
Sit-ups 30 s	20.83 ± 3.55	23.60 ± 4.46	0.001	21.93 ± 3.34	23.43 ± 3.61	0.001

CMJ—Countermovement jump; SJ—Squat jump; 4 × 10—agility test; SAR—Sit and Reach; MB—medicine ball.

## Data Availability

The data presented in this study are available on request from the corresponding author.
